# Protein Composition of the Bovine Herpesvirus 1.1 Virion

**DOI:** 10.3390/vetsci4010011

**Published:** 2017-02-20

**Authors:** Kaley A. Barber, Hillary C. Daugherty, Stephanie E. Ander, Victoria A. Jefferson, Leslie A. Shack, Tibor Pechan, Bindu Nanduri, Florencia Meyer

**Affiliations:** 1 Department of Biochemistry, Molecular Biology, Entomology & Plant Pathology, Mississippi State University, Mississippi State, MS 39762, USA; kaleyabarber@gmail.com (K.A.B.); hecdaugherty@gmail.com (H.C.D.); stephanie.e.ander@gmail.com (S.E.A.); vajefferson77@yahoo.com (V.A.J.); 2 Department of Basic Sciences, College of Veterinary Science, Mississippi State University, Mississippi State, MS 39762, USA; Shack@cvm.msstate.edu (A.S.); BNanduri@cvm.msstate.edu (B.N.); 3 Institute for Genomics, Biocomputing and Biotechnology, Mississippi State University, Mississippi State, MS 39762, USA; pechan@ra.msstate.edu

**Keywords:** bovine herpesvirus, virion, proteomics

## Abstract

Bovine herpesvirus (BoHV) type 1 is an important agricultural pathogen that infects cattle and other ruminants worldwide. Acute infection of the oro-respiratory tract leads to immune suppression and allows commensal bacteria to infect an otherwise healthy lower respiratory tract. This condition is known as the Bovine Respiratory Disease (BRD). BoHV-1 latently infects the host for life and periodical stress events re-initiate BRD, translating into high morbidity and large economic losses. To gain a better understanding of the biology of BoHV-1 and the disease it causes, we elucidated the protein composition of extracellular virions using liquid chromatography-mass spectrometry analysis. We detected 33 viral proteins, including the expected proteins of the nucleocapsid and envelope as well as other regulatory proteins present in the viral tegument. In addition to viral proteins, we have also identified packaged proteins of host origin. This constitutes the first proteomic characterization of the BoHV virion.

## 1. Introduction

Bovine herpesvirus type 1 (BoHV-1) is an important agricultural pathogen in the United States and world-wide. BoHV-1 belongs to the *Herpesviridae* family of viruses within the *Varicellovirus* genus, composed of a double-stranded DNA genome enclosed in an icosahedral nucleocapsid, and surrounded by a host-derived lipid membrane where viral glycoproteins are embedded. BoHV-1.1 largely infects cattle but is increasingly being detected in other ruminants such as domesticated bison and buffalo [1,2,3]. There are three viral subtypes: BoHV-1.1, BoHV-1.2a and BoHV-1.2b that were characterized through endonuclease restriction patterns [4,5]. Most of the BoHV-1.1 isolates are of respiratory origin while BoHV-1.2 are isolated from genital infections. In addition, BoHV-1.1 and 1.2a are also associated with the occurrence of abortions [4,5,6].

The respiratory form of BoHV-1.1 infection initiates in the oro-respiratory mucosa with symptoms including fever, anorexia, coughing, excessive salivation and nasal discharge, conjunctivitis with lacrimal discharge and inflamed nares. The virus replicates and kills epithelial cells of the respiratory mucosa [7], causing extensive epithelial tissue damage and necrosis. The virus can also infect CD4+ T cells [8], impair antigen processing and CD8+ T cell recognition of infected cells [9,10,11], in addition to dampening the host-mounted interferon response using diverse strategies [12,13,14,15,16]. The impaired innate defenses of the host open the opportunity for commensal bacteria of the respiratory tract belonging to the *Pasteurellaceae* family [17] to colonize the otherwise healthy lower respiratory tract and lungs [17]. This multi-factorial and poly-microbial condition is known as the Bovine Respiratory Disease (BRD). BoHV-1.1 constitutes a central factor in the development of this condition because the virus establishes a lifelong latent infection in the sensory neurons, and recurrent stress reactivates the virus into a full-blown acute infection that initiates a new episode of BRD [18,19]. Bacterial secondary infections and pneumonia combined with weight loss, decreased milk yield, and treatment-associated costs due to recurring outbreaks cause significant yearly economic losses to cattle industry [19,20]. In Europe, the Americas this disease is equally worrying [21,22,23,24,25] and severe restrictions exist regarding commerce of seropositive animals [26].

An important first step for better understanding the biology of BoHV-1.1 and the disease it causes is to learn about the basic constituents of the BoHV-1.1 particle. Recent publications have reviewed the body of knowledge gained from the proteomic analysis of isolated herpesvirus virions [27,28]. Proteomic characterization of viral particles allow for the understanding of architecture, the viral strategies for infection and interference of cellular function. It is important to characterize the proteins that are packaged because these proteins are the first to contact the host, and these early interactions may determine the infection outcome. This study characterizes the protein composition of the BoHV-1.1 viral particle using mass spectrometry (MS) analysis of purified extracellular virions. We report a total of 33 viral proteins that make up the viral particle, in addition to a few host proteins. This is the first comprehensive characterization of the protein content of the Bovine herpesvirus 1.1 viral particle.

## 2. Materials and Methods

### 2.1. Cells and Virus

The viral strain used in this study is Bovine herpesvirus type 1.1 Cooper isolate (GenBank Accession number JX898220.1). The cells used to culture the virus were Madin–Darby Bovine Kidney (MDBK) cells grown in Dulbecco’s modified Eagle’s medium supplemented with 5% (v/v) fetal calf serum, 100 micro grams per milliliter (μg/mL) streptomycin and 100 U/mL penicillin, and incubated in a humidified incubator at 37 °C and 5% CO_2_.

### 2.2. Virus Purification

Madin–Darby Bovine Kidney (MDBK) cells were infected with a multiplicity of infection (MOI) of 5 for 48 h. Infected cell supernatants (SN) were centrifuged at 4000 rpm for 30 min at 4 °C. The clarified SN was separated into 25 milliliter (mL) aliquots and spun down through a 30% sucrose cushion in ultracentrifuge for 2 h at 25,000 RPM (110,000× *g*) and resuspended in 1 mL Tris-EDTA buffer (1 M Tris-HCl and 0.2 M Ethylenediaminetetraacetic acid (EDTA)). This process was repeated 10 times, to obtain 10 mL of sucrose-concentrated SN. A DNAse I treatment (50 µg/mL) was carried out for thirty min at 4 °C, after which the mixture was overlayed on a 10% Ficoll cushion and centrifuged at 25,000 RPM for 2 h at 4 °C. The final pellet was resuspended in 1 mL of TE. The entire procedure was repeated with uninfected (or mock-infected) cell culture SN. Virion purification was assessed by negative staining and visualization via Transmission Electron Microscopy. The virus was quantified at various stages of purification by utilizing a standard plaque assay. This was also useful in determining that the mock-infected supernatants did not contain any infectious particles.

Trypsin treatment of purified virions was carried out as described by Zhu and colleagues [29].

### 2.3. Mass Spectrometry Sample Preparation

Protein in mock and BoHV-1.1-infected samples (*n* = 3 for each) was solubilized in a final concentration of 4% sodium dodecyl sulphate (SDS) and treated with a protease inhibitor cocktail (Sigma-Aldrich, Saint Louis, MO, USA). Samples were prepared for quantification with the Thermo Scientific Pierce BCA Protein Assay Kit (Fisher Scientific, Pittsburgh, PA, USA) and protein concentration was determined using a plate spectrophotometer (ThermoMax Microplate, Molecular Devices, Sunnyvale, CA, USA).

One hundred micrograms (µg) of protein from each sample were precipitated with methanol and chloroform (4:1), washed with methanol, spun and air-dried in a desiccator prior to tryptic digestion. Protein samples were treated with 8 M urea at room temperature for 30 min, reduced with 5 mM dithiothreitol at 65 °C for 10 min and alkylated with 10 mM iodoacetamide at 37 °C for 30 min. Protein samples were diluted with water, pH was adjusted to 7.5 and protein was digested with 2 µg of molecular biology grade porcine trypsin (Promega Corporation, Madison, WI, USA) in a 50:1 ratio of protein:trypsin, overnight at 37 °C. Tryptic peptides were cleaned using a strong cation exchange trap (Michrom BioResources Inc., Auburn, CA, USA), eluted in high salt buffer (5 mM NaH_2_PO_4_, 25% acetonitrile, 0.25 M KCl, pH 3) and dried. Dried tryptic peptides were desalted using a peptide macrotrap (Michrom BioResources Inc., Auburn, CA, USA), eluted in 0.1% triflouroacetic acid, 95% acetonitrile solution and air-dried in a desiccator.

### 2.4. Transmission Electron Microscopy

Formvar and carbon-coated 300 mesh copper grids (Electron Microscopy Sciences) were floated on a drop of the purified virus for 10 min. Grids were rinsed briefly by a quick pass on a drop of distilled H_2_O. The grids were then floated on a drop of 2% aqueous uranyl acetate for 30 s. Excess liquid was removed by gently wicking filter paper. Once dry, the grids were examined using a JEOL JEM 1230 Transmission Electron Microscope at 80 kV. Images were acquired and measured using the Advanced Microscopy Techniques Image Capture V700 software (Advanced Microscopy Techniques, Woburn, MA USA). Virion dimensions are expressed as mean ± SEM.

### 2.5. Liquid Chromatography—Tandem Mass Spectrometry and Database Searches 

Spectral data were collected using an Orbitrap LTQ Velos mass spectrometer (Thermo Fisher Scientific, Waltham, MA, USA) linked with an UltiMate 3000 nano flow HPLC system (Thermo Fisher Scientific). A total of two µg of the trypsin-digested protein was loaded on a reversed phase fused silica Acclaim PepMap C18 column, measuring 75 µm × 150 mm (Thermo Fisher Scientific). Peptides were separated and eluted using a constant flow rate of 0.3 microliters per minute in a 60-min long linear gradient of acetonitrile (in 0.1% formic acid): 2%–55% for 35 min, 95% for 10 min, 2% for 15 min. Peptides were detected by a linear trap mass detector, in the data-dependent acquisition mode with dynamic exclusion being applied. Eight scan events were employed: one MS scan (m/z range: 300–2000) followed by seven tandem mass spectrometry (MS/MS) scans for the seven most intense ions detected in the MS scan. Selected parameters were set as follows: Normalized collision energy: 35%; automatic gain control “on” with MSn Target 4 × 104; isolation width (m/z): 1.5; capillary temperature 170 °C; spray voltage 1.97 kV. [29].

The .raw files were searched using the SEQUEST HT algorithm of the Proteome Discoverer 2.1 SP1 software (Thermo Fisher Scientific) with given parameters: Lowest and highest charge: +1 and +3, respectively; minimum and maximum precursor mass: 300 and 6000 Da, respectively; minimum S/N ratio: 3; enzyme: trypsin; maximum missed cleavages: 2; dynamic modifications: cysteine carbamidomethylation (+57.021), methionine oxidation (+15.995) and methionine dioxidation (+31.990).

The spectral data were matched against the target BoHV type 1 protein database downloaded from NCBI (National Center for Biotechnology Information) (www.ncbi.nlm.nih.gov), and *Bos taurus* referenced protein database downloaded from UniProt (Universal Protein Resource) (www.uniprot.org), both as of November 2015. To calculate the false discovery rate (FDR), the software uses the decoy database created by reversing all protein sequences of the target database. Matches were filtered by value of FDR <1.0%. One “hit” identifications were excluded, however proteins identified by at least one unique peptide were accepted, if the peptide was detected multiple times across the replicates. The exponentially modified protein abundance indexes (emPAI), calculated automatically by Proteome Discoverer, were used to approximate the absolute amounts of identified proteins within the sample.

The inclusion/exclusion criterion for host proteins in virion preparations is as follows: host proteins had to appear in at least two of the three samples to be included. At the same time, proteins detected in any of the uninfected supernatants were automatically rejected from the list.

### 2.6. Protein Analysis (Western Blot and Silver Staining)

Twenty μg of whole cell lysates or 5 μg of virion proteins were suspended in 4× Laemmli sample buffer (0.2 mM Tris pH 6.8, 8% SDS, 40% (w/v) glycerol, 0.08% bromophenol blue, 4% β-mercaptoethanol, 0.005 mM EDTA) and boiled for 5 min. After a brief spin, the supernatant was separated by SDS polyacrylamide gel electrophoresis (PAGE) at 150 V for 90 min. Proteins were transferred to a polyvinylidene fluoride membrane via wet electroblotting at 200 mAmps for 90 min, and blocked for 1 h in 5% non-fat dry milk (NFDM) in Tris buffered Saline (TBS) (50 mM Tris pH 7.4, 150 mM NaCl) supplemented with 0.05% Tween-20 (TBS-T). Primary antibodies used were: anti-glycoprotein E (gE) (kindly provided by Shafiql Chowdhury), anti-VP8 (kindly provided by Sylvia Van der Hurk) and anti-tubulin (sc-5286 Santa Cruz Biotechnologies, Dallas, TX, USA), diluted in 5% NFDM in TBS-T and incubated overnight at 4 °C in a rotary platform. Membranes were washed three times with TBS-T at 10-min intervals followed by a 1-h incubation at room temperature with the corresponding horseradish peroxidase-conjugated secondary antibody (anti-goat sc-2020 from Santa Cruz Biotechnologies or anti-mouse #31430 from ThermoFisher (Waltham, MA, USA)) diluted 1:5000 in 5% NFDM in TBS-T. Membranes were washed three times with TBS-T before substrate (SuperSignal West Pico #34080, ThermoScientific (Waltham, MA, USA) was added and chemiluminescence was visualized on film (CL-X Posure^TM^ Film #34091, ThermoScientific (Waltham, MA, USA). Silver staining was performed using Silver Stain Plus (BioRad 161-0449) (Hercules, CA, USA), following the manufacturer’s recommendations.

## 3. Results

### 3.1. Virion Purification and Imaging

**Figure 1:**
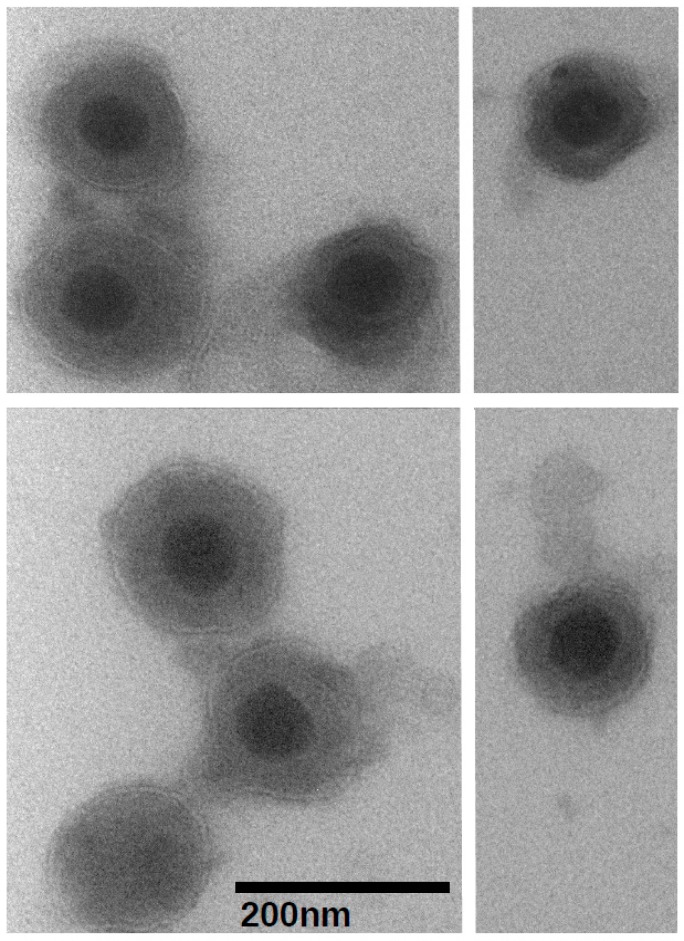
Visualization of purified Bovine herpesvirus type 1.1 virions at 80,000× magnification using the transmission electron microscope and negative staining. The black bar represents 200 nanometers.

With the goal of determining the protein composition of the BoHV-1.1, we developed a virion purification protocol to minimize cellular protein contamination. The purification procedure was monitored throughout using transmission electron microscopy (TEM) and plaque assays to quantitate the virus. Each of three final purified virion samples contained approximately 1 × 10^9^ plaque forming units (data not shown). A set of three equally concentrated mock-infected supernatants were prepared and tested in the same way. Aliquots of concentrated virions were visualized by TEM at various times. Negative staining revealed virion preparations with negligible debris and other cellular fragments. The preparations contained both mature virions as well as some empty envelopes. Figure 1 shows the typical BoHV-1.1 virion preparations as seen under 80,000× magnification. The mock-infected supernatant samples appeared clear, also with little debris. The typical herpesvirus virion morphology was observed as an electron-dense regular-shaped nucleocapsid surrounded by lipid membrane with trilaminar appearance (an electron-lucent space at the center of two electron-dense sheets). Virions in all three biological replicas were measured (total *n* = 103) using the Advanced Microscopy Techniques Image Capture V700 software (Advanced Microscopy Techniques, Woburn, MA, USA), revealing the virion's dimensions. The nucleocapsid measured 68.55 ± 0.58 nm, while the enveloped virions measured an average of 166.0 ± 2.61 nm. The dimensions of the BoHV-1.1 virions are comparable to those of other alphaherpesviruses [30,31].

### 3.2. Viral Proteins Present in the Virion

**Figure 2:**
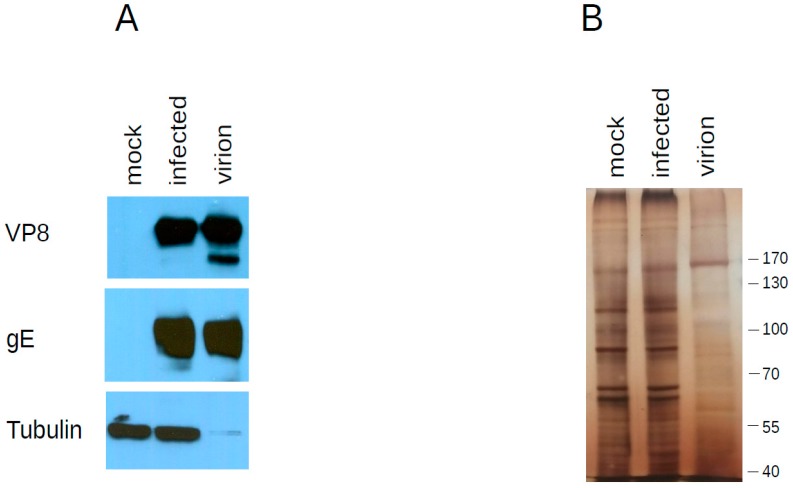
Western blot analysis of proteins found in the virion. (**A**) Twenty micrograms of whole cell lysates of uninfected (mock) and infected cells were separated by SDSPAGE next to 10 micrograms of purified virion extracts. Each blot was probed with the antibody indicated on the left to document its presence in purified virions (**B**) Identical gel as in A was silver stained instead.

Three independent virion preparations were analyzed by mass spectrometry to determine the protein content of the BoHV-1.1 virion. Table 1 summarizes the viral proteins detected and assigns them a known or suspected location based on the description of corresponding open reading frames in herpes simplex type 1 (HSV-1) [32,33]. Overall, we detected 33 viral proteins in the BoHV-1.1 virion. As expected, we identified glycoprotein (g)B, gH, gD, gC, gM, gE, gG, gI and gL, with the exception of gK and gN. Figure 2 shows the presence of gE in virion preparations.

**Figure 3:**
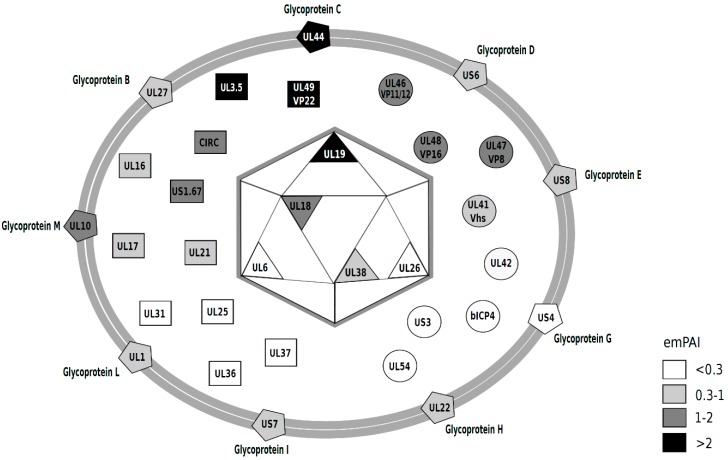
Diagram of the BoHV-1.1 viral particle, representing the viral proteins detected in this study. The relative abundance was estimated based on emPAI scores [39] (Table 1) and are presented on a gray-scale that correlates with abundance (darker = more abundant). emPAI values < 0.3 (white); 0.3–1 (light gray); 1–2 (dark gray) and >2 (black). The diagram was inspired by those of past work [30,31,40].

The nucleocapsid proteins detected were the major capsid protein UL19, the capsid triplex subunits 1 and 2, UL38 and UL18, respectively, as well as the scaffolding protein UL26 and the encapsidation protein UL6. Other proteins are assumed to localize in the tegument or envelope. We have chosen to make an arbitrary classification within tegument proteins that clusters proteins with enzymatic or regulatory activity and comprises tegument proteins such as a catalytic subunit of the DNA polymerase (UL42), bICP4, UL47 (VP8) or UL41 (Vhs), among others (Table 1 and circles in the virion diagram, Figure 3). We further show via Western blot that VP8 is present in purified virions (Figure 2). Among the tegument proteins that we expected to find are UL48 (b-TIF or VP16), UL36 and UL37, as these proteins have long been recognized to be part of the BoHV-1.1 tegument [34] or assumed to be a part of the tegument, as judged by homology to closely related herpesviruses.

After mass spectrometry data acquisition, it is often desirable to quantitate the proteins present in a sample. SILAC-based methods are used for relative quantification in differential expression analyses of viral infection [35,36,37]. A simple method to infer relative protein abundance that does not require isotopic labeling is to analyze the number of spectral counts derived from MS/MS analysis to estimate the relative abundance of each protein in the sample [38]. A method for absolute quantitation also used to determine absolute abundance of proteins is the exponentially modified protein abundance index (emPAI) [39]. This index takes into account how large the protein is because it is expected that larger proteins will produce more tryptic peptides. These methods have been used singly or in combination to quantitate proteins in other herpesvirus virions [30,31,40,41]. Based on emPAI analysis, the most abundant proteins in the BoHV-1.1 virion are UL3.5 followed by the tegument protein VP22, glycoprotein C (UL44), the major capsid protein (UL19) and VP8 (UL47). Table 1 presents the emPAI values for all proteins in the virion, and their abundance is illustrated with the use of a gray-scale in the diagram in Figure 3. In Figure 2, we show the presence of two of these viral proteins in purified virions: an abundant protein (VP8) and one less abundant protein (gE) (based on emPAI scores).

Proteins unique to BoHV-1.1 or a few other herpesviruses found in the virion are Circ, UL1.67 and UL3.5; the latter one is amongst the top most abundant proteins in the virion. Nonstructural proteins detected were DNA processivity subunit (UL42) as well as several regulatory proteins such as bICP4, bICP27, bTIF/VP16 and US3 kinase.

### 3.3. Host Proteins Present in the Virion

By searching the mass spectra against the *Bos Taurus* protein database, we identified a few host proteins present in the virion, similarly to other herpesviruses such as human cytomegalovirus (HCMV) where only one host protein was detected in the virion [42], but in stark contrast to HSV-1, BoHV-4 or Pseudorabies virus (PRV, also known as suid herpesvirus 1 (SuHV-1), where 49, 15, or 48 host proteins have been reported to be packaged in the virions, respectively [30,31,40]. This study found a total of twenty, thirty-one and seven proteins in each of the three virion preparations (forty in all). However, this list was shortened after examining the control (uninfected) supernatant preparations (see the methods section) because host proteins that appeared in virions and in control preparations (Supplementary Tables S1 and S2) were not considered for further study. Histone H4 was detected in all three virion preparations, with a relatively high sequence coverage (29%, data not shown). Interestingly, other histones (H2B type 1 and a variant of H2A (H2A.V) were also detected in two of the three virion samples (Table 2). The criteria for inclusion of host proteins in Table 2 were (1) the protein was present in at least two of the virion preparations and (2) the protein was not present in any of the uninfected controls. In addition to histones, ribosomal proteins L7, L8, L14, and S6 and tubulin beta-5 chain were also detected in two of the three virion preparations (Table 2). As mentioned above, none of these proteins were found in any of the mock preparations. Other host proteins were also retrieved in virion samples, forty in total (Supplementary Table S1). However, thirty-two of them either showed up in mock preparations or were only present in only one of the three virion samples (Supplementary Table S1).

For all herpesviruses for which virion proteomics has been completed, the reported number of host proteins packaged in the tegument varies widely, ranging from only one for the Channel Catfish Virus to about 70 for the [30,31,40,41,43,44]. In our study, we controlled for host proteins that may make it into the final sample as a result of the purification strategy. Therefore, we concentrated uninfected culture supernatants in the same way as we did with infected supernatants. In each of three separate uninfected (mock) preparations, we identified nine, seven and fifteen host proteins (complete list in Supplementary Table S2). A group of four proteins was consistently detected in the mock-preparations: Serum albumin, Pancreatic trypsin inhibitor, Alpha-2-HS-glycoprotein and cytoplasmic Actin 1 (Table 2). In addition, serotransferrin was present in two of the three mock preparations. When examining the virion preparations, four of these proteins (albumin, pancreatic trypsin inhibitor, alpha-2-HS-glycoprotein and serotransferrin) were found in every virion preparation, while cytoplasmic actin appeared in one of them.

## 4. Discussion

As we study the bovine herpesvirus type 1 and the complex respiratory disease in which it participates, a fundamental starting point is to examine the basic composition of the virus. In the present work we provide proteomic evidence of the protein content of the BoHV-1.1 viral particle. We expectedly found most of the glycoproteins and the proteins that compose the nucleocapsid. In BoHV-1, as in other herpesviruses, gC and gD are amongst the first viral proteins to contact the host. Glycoprotein C interacts with heparan-sulfate on the cell surface [45] while gD is known to interact with the nectin-1 receptor in BoHV-1 and in HSV-1 [46,47]. Glycoprotein D is a major target of neutralizing antibody production and naturally a target of extensive vaccine development efforts [48]. Most other viral glycoproteins also localize to the viral envelope [49]. Absent from BoHV-1.1 virions were the membrane protein UL20 and glycoproteins K and N. In HSV-1, UL20 and gK interact and play an important role in intracellular trafficking and viral budding [50,51]. UL20 in HSV-1 and its homolog in PRV are present in the virion [52,53]. While the proteomic analysis of HSV-1 virions detected UL20 only by WB analysis, gK was not detected [30]. Glycoprotein N (UL49.5 gene) has been shown in BoHV-1 to abolish translocation of peptides to the endoplasmic reticulum by the transporter associated with antigen processing (TAP), thereby down modulating MHC class I expression and the immune response [54]. We did not find gN in the virion, and hypothesize that due to its interaction with TAP it is likely that gN is localizes to the ER membrane during infection. The HSV-1 particle also does not seem to have this protein in the mature particle [30].

Three proteins of unclear function and unique to BoHV-1 and few other herpesviruses were detected in the virion. Circ is a small, myristylated protein non-essential for replication in tissue culture [55] that localizes to the virion [56]. The homolog protein in equine herpesvirus-1 (EHV-1), UL1, also localizes in the tegument [57], in contrast to ORF-2 of varicella zoster virus (VZV) which does not [58]. Circ's function is not clear. The US1.67 protein is only encoded in the genomes of BoHV-1, BoHV-5, canid herpesvirus 1 and in most herpesvirus of equine. In EHV-1 the homolog protein is IR6 or V67 and it facilitates the egress of nucleocapsids from the nucleus [59]. US1.67 protein was classified as non-essential for BoHV-1.1 replication in tissue culture [55], consistent with a recent finding of a spontaneous deletion of this gene from the genome of a vaccine strain [60]. The viral UL3.5 gene is present in BoHV-1 [34,61,62], infectious laryngotracheitis virus [63] EHV-1 [64], PRV [65] and VZV [66]. In BoHV-1.1 it appears to be an essential gene [55] while its function to date remains unclear. However, its interaction with the b-TIF/VP16 protein [67] may assign it a role in DNA replication [68]. According to emPAI analysis UL3.5 was the most abundant protein in the viral particle and its role may be more important than previously recognized. In PRV, UL3.5 is involved in cel-to-cell spread because UL3.5 deletion mutants are severely impaired in their spreading to neighboring cells [69]. Other reports associate its function to neurovirulence [70].

Non structural proteins involved in DNA metabolism were not intuitively expected to be present in virions. However, one component of the DNA replication machinery was the DNA polymerase processivity subunit (UL42), which has also been reported to be present in the tegument of human and murine cytomegalovirus [43,71] but not in the HSV-1 virion [30]. Carrying these proteins ready-made in the virion may represent an advantage to quickly engaging in gene expression or replication soon after cell entry. Similar is the case for transcriptional regulatory proteins, and we have detected bICP4 in this study but not bICP0. This is in contrast to HSV-1 where both these proteins ar carried in the virion [30] is assist in immediate-early gene expression.

UL47 (VP8) is a major component of the virion critical for viral replication [72] and participates in the re-distribution of promyelocytic leukemia (PML) protein in the nucleus [73]. In the host cell, VP8 is phosphorylated by host CK2 and viral US3 kinases [74] and mostly the unphosphorylated form of VP8 is packaged in the virion [73]. Expectedly we detect VP8 in virions and infected cells by western blot (Figure 2). A lower-migrating band is enriched in the purified virions with respect to infected cells, suggesting that this may be the unphosphorylated form of VP8. Our MS results also show evidence for the presence of US3 kinase in the BoHV-1.1 virion (albeit at low levels) as is the case for PRV and HSV-1 virions [30,31,75].

The presence of Histone proteins in the BoHV-1.1 virion was not surprising as histone proteins have been detected in the tegument of other herpesviruses: histone 2A in murine cytomegalovirus (MCMV) [43], histones 4 and 2B were found in acelaphine herpesvirus (AlHV-1) virions [76], and histone H4 and histone H2A type 1 were detected in virions of murid herpesvirus 4 (MuHV-4) [44]. In the host, the herpesviral genome is in a highly dynamic stage of chromatinization and it is now established that the degree of chromatinization plays an important role in the regulation of viral gene expression [77,78]. The HSV-1 genome becomes associated with histones as soon as 1 h after infection [79]. It is possible that some herpesviruses carry histone proteins in the tegument to aid in the early steps of chromatinization and gene expression.

The host protein tubulin has also been reported in other herpes virions such as the human cytomegalovirus (HCMV) [41], Kaposi’s Sarcoma-associated herpesvirus (KSHV) [80] and cyprinid herpesvirus 3 (CyHV-3) [81]. HSV-1 infection [82] as well as other herpesviruses [83,84] rely on a functional microtubule network to transport its nucleocapsids to the nucleus upon de-envelopement [82,85]. This dependence has not been formally investigated for BoHV-1.1. Two BoHV-1.1 proteins have been shown to interact with components of the cytoskeleton: US3 kinase induces dramatic rearrangement of the actin cytoskeleton and may directly interact with microtubules [86], while VP22 tegument protein has also been shown to interact with the microtubule network [87]. Both VP22 and US3 were detected in this study. Therefore, the incoming particle may use this network of proteins to interact with microtubules at the very early stages after cell entry. On the other hand, the large tegument protein UL36 [82] and UL37 [88] of HSV-1 are involved in transport of nucleocapsids from the plasma membrane to the nuclear periphery, suggesting that UL36 and UL37 homologs in BoHV-1 (which are present in the virion) may have a similar function of interacting with the cytoskeleton. Therefore carrying tubulin, UL36, UL37, VP22 and US3 in the virion may be advantageous to initiate a prompt transport of nucleocapsids to the nucleus.

The presence of ribosomal proteins is a novel finding of the BoHV-1.1 virion as no other herpesviruses have detected these. The fact that none of the ribosomal proteins were detected in mock preparations supports the notion that they are not a result of a protocol bias. Because this study did not analyze additional protease-treated virion samples via mass spectrometry, we cannot reject the idea that ribosomal proteins may be present outside the virion. The discovery that HCMV carries viral messenger RNA in the virion was a revelation in 2000 [89] followed by similar findings for HSV-1 [90]. The presence of a ribosomal proteins could play a role in guarding the packaged mRNAs to ensure they reach the ribosomes upon viral fusion. The question of whether there is RNA in the BoHV-1.1 particle has not been addressed.

Serum albumin is the second most abundant protein in serum [91] often presenting challenges for proteomic detection of low abundance proteins [92] It was expected that this and other serum proteins such as transferrin could co-purify with virions due to culture conditions, and this *was* the main motivation for the implementation of uninfected (mock) controls for virion preparation. However, the finding that all three mock preparations contained cytoplasmic Actin 1 was not expected since actin is the most reported host protein present in the tegument of herpesviruses [28], followed by proteins in the Annexin family [30,40,42,43,93]. In this study, because mock preparations were used as a negative control, cytoplasmic actin was eliminated from our list of potentially packaged host proteins, as was the case of Annexin A2 (found in one of the mock preparations) (Supplementary Table S1). Overall, from 40 host proteins detected in virion preparations, 10 were shared with mock preparations. The complete list can be seen in the Supplementary Table S1. In conclusion. the presence of host proteins in uninfected SN suggests that even a carefully executed purification procedure will drag host proteins to the final pellet.

The discussion of which cellular proteins are truly associated to the herpesvirus virion has continuously generated debate amongst herpesvirologists [28]. It seems evident that each herpesvirus has a different virion composition, with the greatest protein diversity observed in the tegument. However, the issue remains as to the bias that may be contributed by different purification practices. The choice to control for protocol bias has led us to discover that some proteins consistently make their way into virion preparations (see Table 2, bottom), either due to their abundance (e.g., albumin) or due to protocol (e.g., buffers used, centrifugation). We would like to highlight that none of the host proteins detected in our virion preparations (histones, ribosomal proteins and tubulin) appeared in the mock preparations, suggesting that those proteins are specifically associated with BoHV-1.1 virions.

Through comparative virology we look for similarities in the composition of herpesviruses with the expectation of finding emerging patterns that can help us better understand some of the early events that critically influence the infection outcome. However, herpesviruses infect a variety of hosts and interact with different cells and molecules within the host. Thus, each virus will most certainly carry a distinctive set of proteins in the virion to aid in this task. In this work we have elucidated for the first time the unique set of viral and host proteins that compose the BoHV-1.1 virion. We hope that this work will contribute to future studies that complement the knowledge of the viral particle, such as determining whether or not BoHV-1.1 packages any messenger RNA.

## Figures and Tables

**Table 1:** Viral proteins detected in purified BoHV-1.1 virions.

BHV-1.1 Protein	Description/Alternative Name	Function	Size (aa)	MW (kDa)	# of Peptides ^a^	# of Spectra ^a^	# Unique Peptides ^a^	% Coverage	emPAI	NCBI Accession
Capsid										
UL6	Capsid portal protein	DNA encapsidation	692	75	4	12	4	6.4	0.245	AFV53413
UL18	Capsid triplex subunit 2	Capsid morphogenesis	316	33.2	7	34	7	24.1	1.637	AFV53401
UL19	Major capsid protein	Capsid morphogenesis	1389	150	32	174	32	26.0	2.073	AFV53400
UL26	Capsid scaffolding protein	Scaffold/serine protease	622	63.7	2	11	2	4.5	0.274	AFV53392
UL38	Capsid Triplex Subunit 1	Capsid morphogenesis	475	50	4	14	4	12.8	0.374	AFV53380
Envelope										
UL1	Glycoprotein L, gL	Cell entry/cell-to-cell spread	158	17	3	12	3	17.7	0.874	AFV53419
UL10	Glycoprotein M, gM	Virion morphogenesis/membrane fusion	411	42.4	5	26	5	11.7	1.683	AFV53409
UL22	Glycoprotein H, gH	Cell entry/cell-to-cell spread	842	88.3	9	44	9	11.3	0.688	AFV53397
UL27	Glycoprotein B, gB	Cell entry/cell-to-cell spread	928	101.9	13	83	1	14.8	0.778	AFV53391
UL44	Glycoprotein C, gC	Cell attachment	521	55.4	13	113	13	29.9	3.786	AFV53374
US4	Glycoprotein G, gG	Cell-to-cell spread	444	46.6	1	9	1	3.4	0.116	AFV53429
US6	Glycoprotein D, gD	Cell attachment	417	44.9	3	35	3	9.4	0.551	AFV53430
US7	Glycoprotein I, gI	Cell-to-cell spread	382	39.6	3	12	3	9.4	0.389	AFV53431
US8	Glycoprotein E, gE	Cell-to-cell spread	575	61.2	3	22	3	7.3	0.413	AFV53432
Tegument										
UL3.5	protein V57	Cell-to-cell spread	126	13.4	6	77	6	56.3	6.499	AFV53416
Circ	Myristylated tegument protein	Unknown	246	26.1	4	14	4	24.0	1.512	AFV53363
UL16	Tegument protein UL16	Possible virion morphogenesis	343	36.4	6	29	6	20.1	0.823	AFV53404
UL17	DNA Packaging Tegument Protein	DNA encapsidation/capsid transport	703	72.6	7	33	7	11.7	0.455	AFV53403
UL21	Tegument protein	Virion morphogenesis	578	60.2	5	23	5	9.5	0.433	AFV53398
UL25	DNA Packaging Tegument Protein	DNA encapsidatoin	598	63	3	9	3	7.2	0.184	AFV53394
UL31	Nuclear egress lamina protein	Nuclear egress	376	40.9	1	2	1	2.1	0.116	AFV53387
UL36	Large tegument protein	Capsid transport	3291	336	10	47	10	3.4	0.147	AFV53382
UL37	Tegument protein	Virion morphogenesis	1034	106	7	22	7	8.1	0.254	AFV53381
UL49	Tegument protein VP22	Virion morphogenesis	258	26.8	8	106	8	43.0	6.356	AFV53370
US1.67		Unknown	243	27.1	4	16	4	15.6	1.154	AFV53426
Tegument (enzymatic and regulatory proteins)										
bICP4	IE transactivator protein	Transcriptional regulator	1386	141	2	2	2	1.7	0.032	AFV53424
UL41	Virion host shutoff protein, Vhs	Cellular mRNA degradation	459	50	5	28	5	11.3	0.557	AFV53377
UL42	DNA polymerase processivity subunit	DNA replication	408	42.6	3	11	3	8.1	0.28	AFV53376
UL46	Tegument protein VP11/12	Possible gene regulation	748	78.6	11	86	11	22.9	1.572	AFV53373
UL47	Tegument protein VP8	Possible gene regulation	741	80.5	13	132	13	18.2	1.88	AFV53372
UL48	Trans-inducing factor bTIF/VP16	Gene regulation/virion morphogenesis	507	53.1	6	42	6	10.1	1.154	AFV53371
UL54	Multifunctional regulator, bICP27	Gene regulation; RNA metobolism	400	43.3	1	3	1	2.3	0.11	AFV53364
US3	US3 kinase	serine/threonine kinase	468	50.2	1	3	1	3.8	0.101	AFV53428

BoHV-1.1, Bovine herpesvirus type 1.1; MW, molecular weight, emPAI, exponentially modified protein abundance index; NCBI, National Center for Biotechnology Information; **^a^** across biological replicates.

**Table 2:** Host proteins detected in purified virions. Proteins shown appeared in two or three out of the three virion preparations. The complete list of proteins can be seen in the Supplementary Materials.

Host Protein	UniProt Accession	# of Positive Samples	# of Peptides ^a^
Proteins detected in purified virions			
Histone H4	P62803	3	9,9,1
Histone H2A.V	Q32LA7	2	2,1,0
Histone H2B type 1	P62808	2	1,1,0
60S ribosomal protein L7	Q58DT1	2	0,1,1
60S ribosomal protein L8	Q3T0S6	2	3,1,0
60S ribosomal protein L14	Q3T0U2	2	3,1,0
40S ribosomal protein S6	Q5E995	2	2,2,0
Tubulin beta-5 chain	Q2KJD0	2	3,3,0
Proteins that appeared in both virions and mock-infected preparations			
Serotransferrin	Q29443	3	1,1,1
Serum albumin	P02769	3	7,3,2
Pancreatic trypsin inhibitor	P00974	3	5,6,3
Alpha-2-HS-glycoprotein	P12763	3	1,8,1
Annexin A2 *	P04272	2	1,2,0

**^a^** in each of three biological replicates; * Two of the mock samples contained other proteins in this family (Annexin A1 and A5).

## Supplementary Materials

The following are available online at www.mdpi.com/2306-7381/4/1/11/s1, Table S1: Complete list of host proteins found in virion preparations; Table S2: Complete list of host proteins found in mock preparations.

Supplementary File 1
