# Supplementary material for: Protein Composition of the Bovine Herpesvirus 1.1 Virion
Source: Vet Sci. 2017 Feb 20;4(1):11. doi: 10.3390/vetsci4010011 (PMC5606624; doi:10.3390/vetsci4010011)
Supplement: Supplementary File 1 [file vetsci-04-00011-s001.pdf]

veterinary sciences

MDPI

Supplementary Materials: Protein Composition of the Bovine Herpesvirus 1.1 Virion

Kaley A. Barber, Hillary C. Daugherty, Stephanie E. Ander, Victoria A. Jefferson, Leslie A. Shack, Tibor Pechan, Bindu Nanduri and Florencia Meyer *

Table S1. Host proteins detected in purified virions. Below the table, a Venn diagram depicting the results. Red entries are proteins that also appeared in mock-preparations.

Host Proteins in Virion Preparations	Gene Symbol	UniProt Accession	# of positive samples	# of peptides[a]
Histone H4	H4	P62803	3	9,9,1
40S ribosomal protein S6	RS6	Q5E995	2	2,2,0
60S ribosomal protein L14	RL14	Q3T0U2	2	3,1,0
60S ribosomal protein L7	RL7	Q58DT1	2	0,1,1
60S ribosomal protein L8	RL8	Q3T0S6	2	3,1,0
Histone H2A.V	H2AV	Q32LA7	2	2,1,0
Histone H2B type 1	H2B1	P62808	2	1,1,0
Tubulin beta-5 chain	TBB5	Q2KJD0	2	3,3,0
Serotransferrin	TRFE	Q29443	3	1,1,1
Serum albumin	ALBU	P02769	3	7,3,2
Pancreatic trypsin inhibitor	BPT1	P00974	3	5,6,3
Alpha-2-HS-glycoprotein	FETUA	P12763	3	1,8,1
Annexin A2	ANXA2	P04272	2	1,2,0
Actin, cytoplasmic 1	ACTB	P60712	1	0,5,0
Excitatory amino acid transporter 1	EAA1	P46411	1	0,1,0
Fibronectin	FINC	P07589	1	0,3,0
Phosphate carrier protein, mitochondrial	MPCP	P12234	1	0,1,0
Probable tubulin polyglutamylase TTLL1	TTLL1	Q0VC71	1	0,3,0
40S ribosomal protein S8	RS8	Q5E958	1	6,0,0
40S ribosomal protein S9	RS9	A6QLG 5	1	1,0,0
60S acidic ribosomal protein P0	RLA0	Q95140	1	3,0,0
60S ribosomal protein L13	RL13	Q56JZ1	1	1,0,0
Alpha-1-syntrophin	SNTA1	Q0P5E6	1	0,1,0
Annexin A1	ANXA1	P46193	1	0,3,0
Annexin A5	ANXA5	P81287	1	0,1,0
Beta-1,4-glucuronyltransferase 1	B4GA1	Q5EA01	1	0,2,0
Dynein intermediate chain 1, axonemal	DNAI1	Q32KS2	1	0,1,0
Elongation factor 1-alpha 1	EF1A1	P68103	1	0,1,0
Gap junction alpha-4 protein	CXA4	A4IFL1	1	1,0,0
Glyceraldehyde-3-phphate dehydrogenase	G3P	P10096	1	0,1,0
Heat shock cognate 71 kDa protein	HSP7C	P19120	1	0,1,0
Heat shock protein HSP 90-beta	HS90B	Q76LV1	1	0,1,0
Histone H1.2	H12	P02253	1	3,0,0

Insulin-like growth factor 1 receptor (Fragment)	IGF1R	Q05688	1	0,0,1
Melanoma inhibitory activity protein 3	MIA3	Q0VC16	1	0,1,0
Mitochondrial fission regulator 1-like	MFR1L	Q3ZBW 7	1	1,0,0
NEDD8-conjugating enzyme Ubc12	UBC12	A3KN22	1	2,0,0
Ras-related protein Rab-1B	RAB1B	Q2HJH2	1	0,1,0
Serine/threonine-protein kinase 10	STK10	E1BK52	1	0,1,0
Sodium/potassium-transporting ATPase subunit alpha-1	AT1A1	Q08DA1	1	0,1,0

Red print indicates proteins that were identified in mock preparations; **a** in each of three biological replicates.

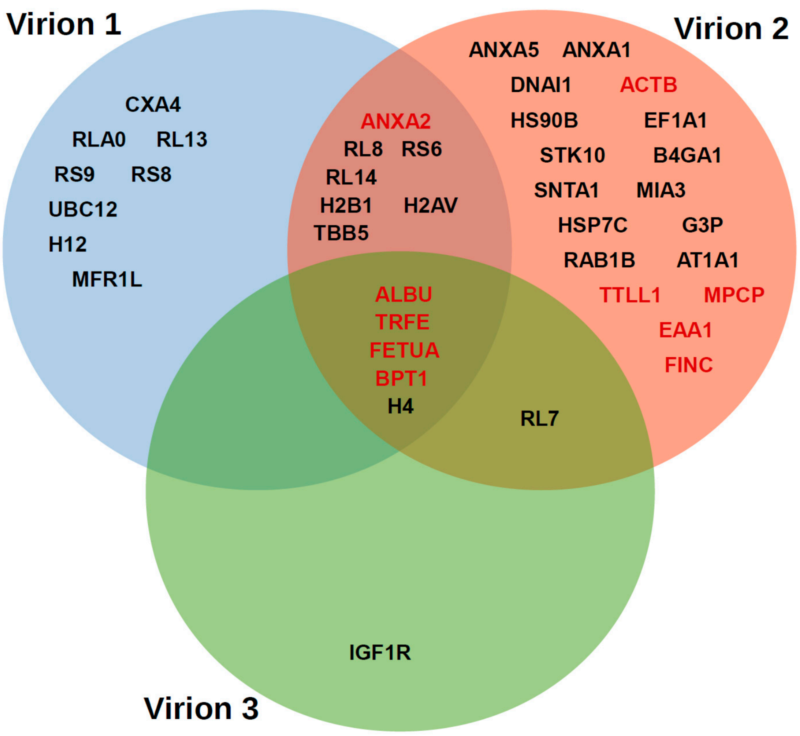

Table 2. Host proteins detected in mock-infected preparations. Below the table, a Venn diagram depicting the results.

Host Proteins in Mock Preparations	Gene Symbol	UniProt Accession	Number of Positive Samples	Number of Peptides[a]
Serum albumin	ALBU	P02769	3	17,6,5
Alpha-2-HS-glycoprotein	FETUA	P12763	3	6,1,2
Pancreatic trypsin inhibitor	BPT1	P00974	3	3,5,6
Actin, cytoplasmic 1	ACTB	P60712	3	1,1,3
Serotransferrin	TRFE	Q29443	2	1,1,0
Annexin A2	ANXA2	P04272	1	0,0,7
Probable tubulin polyglutamylase TTLL1	TTLL1	Q0VC71	1	3,0,0
Excitatory amino acid transporter 1	EAA1	P46411	1	2,0,0

GrpE protein homolog 2, mitochondrial	GRPE2	Q0P5N5	1	1,0,0
Aspartate beta-hydroxylase domain-containing protein 1	ASPH1	A1L515	1	1,0,0
Target of rapamycin complex subunit LST8	LST8	Q17QU5	1	0,1,0
Long-chain fatty acid transport protein 1	S27A1	Q3ZKN0	1	0,1,0
Alpha-2-macroglobulin	A2MG	Q7SIH1	1	0,0,1
Fibronectin	FINC	P07589	1	0,0,2
Heat shock-related 70 kDa protein 2	HSP72	P34933	1	0,0,1
Lactadherin	MFGM	Q95114	1	0,0,1
Peptidoglycan recognition protein 1	PGRP1	Q8SPP7	1	0,0,1
Nuclear pore complex protein Nup85	NUP85	Q3ZC98	1	0,0,1
Phosphate carrier protein, mitochondrial	MPCP	P12234	1	0,0,1
DNA (cytosine-5)-methyltransferase 1	DNMT1	Q24K09	1	0,0,1
Methionine--tRNA ligase, mitochondrial	SYMM	A6H7E1	1	0,0,1
Junctional protein associated with coronary artery disease homolog	JCAD	A2VE02	1	0,0,1

[a] in each of three biological replicates.

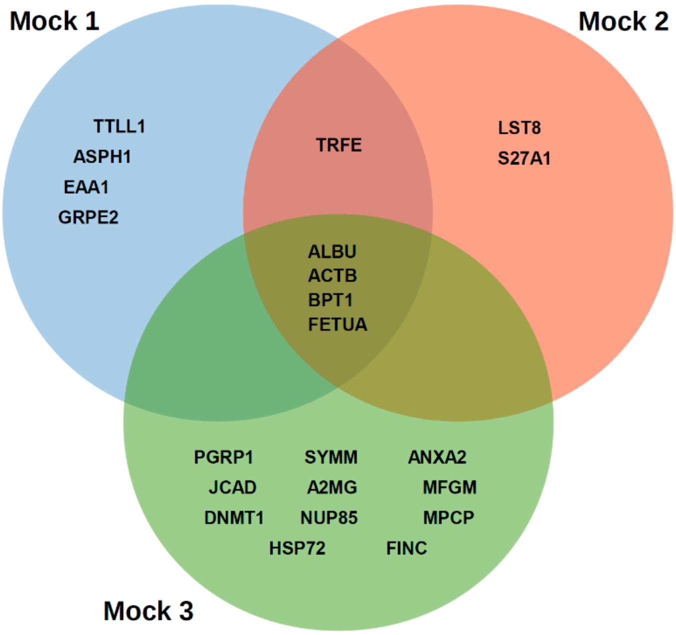

© 2017 by the authors; licensee MDPI, Basel, Switzerland. This article is an open access article distributed under the terms and conditions of the Creative Commons by Attribution (CC-BY) license (http://creativecommons.org/licenses/by/4.0/).
